# Parenteral artesunate compliance and hospital outcomes in children under five with suspected severe malaria in Sierra Leone

**DOI:** 10.5588/pha.25.0047

**Published:** 2026-05-18

**Authors:** A.R.Y. Kamara, I.F. Kamara, P. Thekkur, A.M. Falama, M. Sillah-Kanu, W.K. Lahai, O.O. Fiona, A. George, B.D. Fofanah, N. Sesay, S.M. Tengbe, L. Farma-Grant, A. Bah, F. Thomas, Y.S. Tejan, J.S. Kanu, S. Lakoh, M. Mustapha, R. Zachariah, K. Chandrasekharan Prathija, S.M. Kenneh

**Affiliations:** 1 National malaria Control Programme, Ministry of Health, Freetown, Sierra Leone;; 2 World Health Country Office, Freetown, Sierra Leone;; 3 Centre for Operational Research, International Union Against Tuberculosis and Lung Disease, Paris, France;; 4 Ministry of Health, Freetown, Sierra Leone;; 5 College of Medicine and Allied Health Sciences, University of Sierra Leone, Freetown, Sierra Leone;; 6 KYM Consultancy Ltd, Freetown, Sierra Leone;; 7 UNICEF, UNDP, World Bank, WHO Special Programme for Research and Training in Tropical Diseases (TDR), Geneva, Switzerland.

**Keywords:** SORT IT, Universal Health Coverage, Sustainable Development Goals, malaria elimination, operational research

## Abstract

**OBJECTIVES:**

To assess compliance with national malaria guidelines for confirmatory diagnosis and parenteral artesunate administration, and to evaluate hospital exit outcomes among children under five admitted with suspected severe malaria.

**DESIGN:**

A cohort study utilising routinely collected data in Ola During Children’s Hospital, a tertiary paediatric hospital in Freetown, Sierra Leone, from January to December 2024.

**RESULTS:**

Of 735 admitted children (55% male), 657 (89%) underwent rapid diagnostic tests, but none had microscopic confirmation. A total of 650 (88%) received all three recommended doses of parenteral artesunate at 0, 12, and 24 h. Among the remaining 85 children, 48 (56%) received one or two doses, and 37 received none. Two children died before completion of 24-h dosing schedule. Correct weight-based dosing and scheduling were administered in 420 children (parenteral artesunate compliance = 57% [420/733]). Among admissions, 642 (87%) were discharged and 73 (10%) had unfavourable hospital exit outcomes, including 59 deaths, 13 discharged against medical advice, and 1 absconded. Underweight status, multiple convulsions, and failure to receive correct artesunate dosing were associated with unfavourable hospital exit outcomes.

**CONCLUSION:**

Compliance with recommended parenteral artesunate dosing was suboptimal, potentially contributing to unfavourable hospital outcomes. Strengthened monitoring, mentorship, and enforcement of malaria management guidelines are urgently needed.

Malaria continues to be a major global public health challenge. In 2023, an estimated 263 million cases were reported across the 85 malaria-endemic countries, with sub-Saharan Africa carrying the highest burden, accounting for an estimated 94% of malaria cases worldwide.^1^ Sierra Leone lies within the malaria-endemic belt and experiences intense year-around malaria transmission. According to a 2021 malaria survey, 22% of children under five tested positive for malaria parasites.^2^ In 2024, the country recorded 2,026,084 uncomplicated malaria cases in the under-five, with 2.7% progressing to severe malaria. Malaria accounts for approximately 38% of outpatient visits and 47% of hospital admissions nationwide (all ages).^2,3^ Malaria treatment is critical in children under five as their progression from uncomplicated to severe malaria, and potentially death, can occur rapidly.^4^ Guidelines for malaria management emphasise the need for prompt diagnosis and effective treatment of suspected severe malaria cases.^3,5^ Specifically:

1. Any acute febrile illness with severe manifestations (persistent vomiting, inability to drink or breastfeed, extreme weakness, or convulsions) should be considered suspected severe malaria and urgently referred.
2. At the hospital level, confirmation should be done using a rapid diagnostic test (RDT) and ideally, also with microscopy.
3. Children <20 kg should receive parenteral artesunate 3.0 mg/kg, and those ≥20 kg receive 2.4 mg/kg, administered intravenously or intramuscularly at admission, at 12 h, and at 24 h. Once oral intake is possible, switch to a full 3-day course of oral artemether–lumefantrine or artesunate–amodiaquine.

In line with national guidelines, any child presenting with one or more severe clinical manifestations is classified as having suspected severe malaria and requires immediate emergency management, even before ideal confirmatory microscopy test results are available. There is growing concern about non-compliance with these core practices among health care providers due to frequent staff turnovers, inadequate training, high patient loads, and limited financial resources at health facilities. Non-compliance with treatment guidelines can compromise the quality of malaria care and lead to suboptimal treatment outcomes.^6^ Only a limited number of studies from sub-Saharan Africa have assessed compliance with all components of the WHO severe malaria management guidelines in children, with one study from Uganda reporting compliance as low as 3%.^4^ There are currently no published studies from Sierra Leone that have evaluated compliance to malaria treatment guidelines in under-five children admitted with suspected severe malaria. Such information would be instrumental in guiding policy decisions, improving clinical practices, and strengthening health care delivery systems.

We aimed to assess compliance with two core components of the national malaria treatment guidelines (confirmatory diagnosis and administration of parenteral artesunate) and evaluate hospital exit outcomes among children (under five) admitted for suspected severe malaria at the Ola During Children’s Hospital (ODCH) in 2024. We determined the:

- Clinical manifestations of suspected severe malaria.
- Proportion tested with an RDT, microscopy, or both.
- Proportion who received three doses of parenteral artesunate within the first 24 h of admission in the recommended dosage and schedule.
- Hospital exit outcomes and factors associated with unfavourable outcomes.

## METHODS

This was a non-concurrent cohort study utilising routinely collected hospital data.

### Setting

Sierra Leone has a population of approximately 8 million inhabitants.^7^ The country lies within the malaria-endemic belt and experiences intense year-around transmission that peaks mostly at the beginning and end of the raining seasons.^8^ *Plasmodium falciparum* accounts for over 90% of malaria cases in the country.^3,5^ The health system is tiered into primary, secondary, and tertiary levels, with severe malaria cases referred to higher-level facilities for management.^8^ The ODCH is a university-affiliated tertiary paediatric hospital, located in Freetown. It has 164 beds and provides outpatient, emergency, intensive care, neonatal, and inpatient paediatric services.

### National Malaria Control Programme (NMCP)

The NMCP is a division within the Ministry of Health under the Directorate of Disease Prevention and Control. The NMCP is responsible for planning, implementing, and monitoring malaria control activities. It is structured into seven thematic areas: 1) Case management – diagnosis and treatment, 2) Preventive treatment, 3) Surveillance, monitoring and evaluation, and operational research, 4) Integrated vector management, 5) Advocacy, communication and social mobilisation, 6) Procurement and supply chain management, and 7) Programme management.^3^ The Government of Sierra Leone considers a reduction in under-five mortality as a strong indicator of the effectiveness of malaria control efforts.^9^

### Management of suspected severe malaria at ODCH

The management of suspected severe malaria follows the national guidelines.^3^ Children with suspected malaria are brought in directly by their parents/guardians/family members to the outpatient department or are referred from other health facilities. On arrival, children undergo triage using a standardised checklist and those suspected of severe malaria are sent to a medical officer for further assessment. The presence of one (or more) severe manifestation is enough to classify the child as suffering from suspected severe malaria which is considered a medical emergency.^3,4^ The emergency management includes supportive care and the administration of parenteral artesunate. At the hospital level, all children with suspected severe malaria should ideally have parasitological confirmation as this is useful in terms of prognosis, identification of malaria types, and response to treatment.^3^

### Study population and period

The study population included all under-fives admitted to ODCH with suspected severe malaria. The study period was January to December 2024.

### Data source and variables

The data source was individual patient files. Data variables included: unique patient identifier, period of hospitalisation, demographic characteristics including nutrition status, clinical manifestations of suspected severe malaria, type of malaria confirmatory test used, parenteral artesunate dosing and schedule, and hospital exit outcomes.

### Data collection and validation

Data were extracted from individual patient records and entered into Epicollect5 (five.epicollect.net) at the time of record review using a structured data form. The principal investigator validated data entry bi-monthly by cross-checking with a sample of patient records.

### Statistical analysis

Data from Epicollect5 were exported and analysed using Stata version 16 (StataCorp, USA). Descriptive statistics (numbers and proportions) were used to summarise the socio-demographic and clinical characteristics, use of diagnostic tests, and parenteral artesunate administration. Hospital exit outcomes were standardised. Hospital discharge after clinical stabilisation was considered a proxy for favourable treatment outcome. Unfavourable hospital exit outcomes included death, discharge against medical advice, and absconded. The median with interquartile range (IQR) was used to summarise the duration of hospital stay. Unadjusted and adjusted generalised binomial models using modified Poisson regression were used to assess socio-demographic, clinical, and laboratory parameters associated with the unfavourable hospital exit outcomes. Unadjusted relative risk and adjusted relative risk with 95% confidence interval were used to assess measures of association.

### Ethical statement

Ethics approval was received from the Sierra Leone Ethics and Scientific Review Committee (SLESRC – 013/02/2025), which granted a waiver of informed consent in accordance with national regulations for studies using anonymised routinely collected data. The hospital management committee authorised the study. All personal identifiers were removed prior to analysis.

## RESULTS

A total of 735 under-five children (55% male) were admitted with suspected severe malaria. The Figure shows admission peaks in June and October, coinciding with the rainy season.

**FIGURE.**
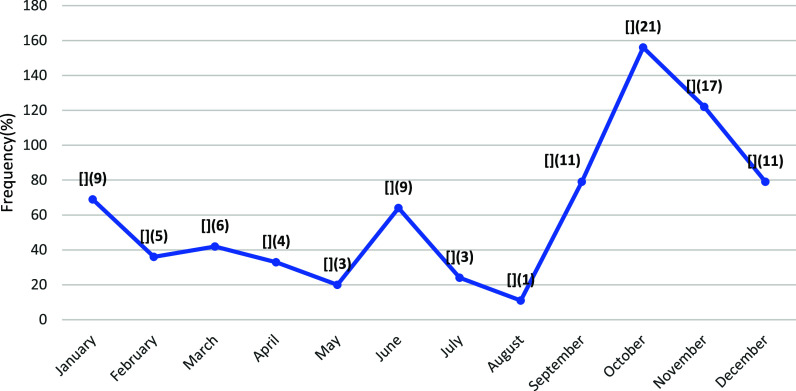
Month-wise distribution of hospital admission among under-five children with suspected severe malaria to Ola During Children’s Hospital (January to December 2024).

Table 1 summarises the demographic, clinical, and laboratory characteristics of children with suspected severe malaria. The majority of children (78%) were below two years of age, with 32% malnourished (underweight). The predominant severe clinical features were: failure to feed with associated vomiting (62%), deep breathing (38%), multiple convulsions (33%), and prostration (26%). The only laboratory test done on admission was blood sugar in 14% of children.

**TABLE 1.** Demographic, clinical, and laboratory findings among under-five children with suspected severe malaria admitted to Ola During Children’s Hospital, January to December 2024.

Characteristics	N (%)^A^
Total	735 (100)
Age (in months)	
0–12	365 (49.7)
13–24	206 (28)
25–36	93 (12.7)
37–48	51 (6.9)
49–60	20 (2.7)
Gender	
Male	407 (55.4)
Female	328 (44.6)
Nutritional status (weight for age^B^)	
Overweight	32 (4.4)
Normal weight	471 (64.1)
Underweight	138 (18.8)
Severely underweight	94 (12.8)
Clinical findings at admission^C^	
Failure to feed with associated vomiting	455 (61.9)
Multiple convulsions (fits)	242 (32.9)
Deep breathing	281 (38.2)
Prostration	189 (25.7)
Pulmonary oedema	21 (2.9)
Clinical jaundice	10 (1.4)
Signs of haemoglobinuria (dark urine)	9 (1.2)
Abnormal spontaneous bleeding	6 (0.8)
Impaired consciousness or unarousable coma.	2 (0.3)
Laboratory findings on admission	
Blood sugar	
Not done	635 (86.4)
Done	100 (13.6)
Hypoglycaemia (blood sugar < 2.2 mmol)	0 (0)
Blood lactate	
Not done	735 (100)
Serum creatinine	
Not done	735 (100)
Urine blood cells	
Not done	735 (100)

A Column percentage.

B Cut-off based on WHO Z score for weight for age.

C Multiple manifestations are possible, and count doesn’t add up to the total.

Among the 735 children admitted with suspected severe malaria, 657 (89% diagnostic compliance) had undergone RDTs, of whom 639 (97%) tested positive for malaria (Table 2). None received a microscopic confirmation of diagnosis. Among the 639 RDT-positive children, 399 (62.4%) received correct parenteral artesunate dosing, while 240 (37.6%) received incorrect dosing and/or schedule. Of the 735 admitted children, 650 (88%) received the complete three-dose regimen of parenteral artesunate administered at 0, 12, and 24 h. Among the remaining 85 patients, 48 (56%) received only one or two doses, and 37 did not receive artesunate at all (reasons for these omissions were not recorded). Two children who died within 24 h were excluded from dosing compliance analysis (n = 733). The correct three-dose regimen and weight-based dosage were administered to 420 children, corresponding to an overall parenteral artesunate treatment compliance of 57% (420/733).

**TABLE 2.** Proportion undergoing rapid diagnostic testing, microscopy, and treatment among under-five children with suspected severe malaria admitted to Ola During Children’s Hospital (January to December 2024).

Variables	n (%)^A^
Total	735 (100)
Rapid diagnostic test	
Not done	45 (6.1)
Done before admission	655 (89.1)
Done after admission	2 (0.3)
Not recorded	33 (4.5)
Positive among tested (N = 657)	639 (97.3)
Microscopy for *P. falciparum*	
Not done	735 (100)
Administration of parenteral artesunate^B^	
Not administered	37 (5)
At 0 h of admission	682 (92.8)
At 12 h of admission	685 (93.2)
At 24 h of admission^C^	665 (90.7)
Received all the three doses at 0, 12, and 24 h^C^	650 (88.7)
Received all the three doses at correct dosage^C^^,^^D^	420/733 (57.3)

A Column percentage.

B Administration of parenteral artesunate is at 0, 12, and 24 h: 0 h refers to administration of the first dose of parenteral artesunate, with the second and third doses given 12 and 24 h after the first dose, respectively.

C Two children who died before 24 h of admission were excluded from the denominator (n = 733).

D Correct dosage is 3 mg/kg body weight/dosage for children with weight <20 kg and 2.4 mg/kg/dosage for children ≥20 kg or over.

The median duration of hospital stay was 3 days (IQR 2–6 days). Table 3 summarises the hospital exit outcomes. Among the 735 children admitted, 642 (87%) were discharged and 73 (10%) had unfavourable outcomes (discharged against medical advice, absconded, and died). Exit outcomes were unrecorded in 20 cases. There were 59 deaths, of whom 37 (63%) did not receive parenteral artesunate in accordance with recommended dosing and schedule (16 received all three doses at incorrect dosages, 12 received fewer than three doses, and 9 did not receive any dose).

**TABLE 3.** Hospital exit outcomes among under-five children with suspected severe malaria admitted to Ola During Children’s Hospital (January to December 2024).

Hospital exit outcomes	n (%)^A^
Total admissions	**735 (100)**
Discharged	642 (87.3)
Discharge against medical advice	13 (1.8)
Absconded	1 (0.1)
Died	59 (8)
Unrecorded	20 (2.7)

A Column percentage.

Significance of the bold: Among the 735 admissions, 642 were children (87.3%), and the majority outcome was discharged alive, which is by far the largest proportion compared to other exit outcomes. A high discharge rate serves as an indicator of case management success, suggesting that most children admitted with suspected severe malaria received effective treatment and recovered sufficiently to leave the hospital.

In multivariable analysis, underweight status (malnutrition), multiple convulsions, and failure to receive the complete three-dose regimen of parenteral artesunate at 0, 12, and 24 h were significantly associated with unfavourable hospital exit outcomes (Table 4).

**TABLE 4.** Factors associated with unfavourable hospital exit outcomes among under-five children with suspected severe malaria admitted at Ola During Children’s Hospital, January to December 2024.

Characteristics	Total	Unfavourable outcome^A^	RR (95% CI)	aRR (95% CI)^B^
Total	715^C^	**N (%)^D^**		
Age (in months)				
0–12	356	50 (14)	1 (0.7–1.3)	1.0 (0.6–1.5)
13–24	201	17 (8.5)	1 (0.7–1.4)	1.0 (0.7–1.6)
25–36	88	2 (2.3)	1 (0.7–1.4)	1.0 (0.7–1.8)
37–48	50	3 (6)	1 (0.7–1.4)	1.0 (0.6–1.6)
49–60	20	1 (5)	1	1.0
Gender				
Male	396	36 (9.1)	0.8 (0.5–1.2)	1.1 (1.0–1.2)
Female	319	37 (11.6)	1	
Nutritional status				
Normal weight	459	37 (8.1)	1	
Underweight	224	30 (13.4)	1.5 (1.1–2.3)	**1.7 (1.1–2.5)**
Overweight	32	6 (18.8)	1.8 (0.8–3.9)	1.7 (0.7–4)
Clinical manifestations at admission^E^				
Prostration	180	18 (10)	1 (0.6–1.7)	1.4 (1.0–2.3)
Failure to feed with vomiting	441	49 (11)	0.8 (0.5–1.3)	1.2 (0.9–1.8)
Multiple convulsions (fits)	237	36 (15.2)	0.5 (0.3–0.8)	**1.6 (1.1–2.5)**
Deep breathing	271	36 (13.3)	0.6 (0.4–1)	1.5 (1–2.3)
Others^F^	29	2 (6.9)	0.7 (0.2–2.6)	0.7 (0.2–2.4)
Rapid diagnostic test				
Positive	625	54 (8.6)	1.8 (0.6–5.3)	1.5 (0.3–3.5)
Negative	18	3 (15.8)		
Received all three doses at 0, 12, and 24 h				
Yes	634	51 (8)	0.3 (0.2–0.5)	
No	81	22 (27.2)		**2.5 (1.5–4.1)**

RR = relative risk; aRR = adjusted relative risk; CI = confidence interval.

A Unfavourable hospital exit outcomes include died, absconded, left against medical advice.

B Generalised binomial model using modified Poisson regression.

C Twenty children with unrecorded hospital exit outcomes were excluded from regression analysis (analytical sample n = 715).

D Row percentage.

E Not having the clinical manifestation is the reference category.

F Others include having one or more of the following symptoms-Clinical Jaundice, dark urine, spontaneous bleeding and pulmonary oedema.

The bold value indicates statistically significant associations with unfavourable hospital exit outcome (*P* < 0.05) among children under five years admitted with suspected severe malaria. Underweight children (aRR 1.7, 95% CI: 1.1–2.5), those presenting with multiple convulsions (aRR 1.6, 95% CI: 1.1–2.5), and those exhibiting deep breathing (aRR 1.5, 95% CI: 1.0–2.3) were significantly more likely to experience poor outcomes. Notably, children who did not receive all three treatment doses at 0, 12, and 24 h faced a substantially higher risk (aRR 2.5, 95% CI: 1.5–4.1).

## DISCUSSION

This study assessed compliance with suspected severe malaria management in children admitted to a tertiary hospital in Sierra Leone. Overall, 87% of children were discharged and considered to have favourable hospital exit outcomes. Nearly one third were malnourished, a factor significantly associated with unfavourable outcomes. RDT diagnostic compliance was 89%, while compliance with parenteral artesunate treatment recommendations was 57%. About one third received incorrect or incomplete doses, and a subset of children admitted with suspected severe malaria did not receive parenteral artesunate, representing ‘missed opportunities’ for life-saving care.

The study findings are of significant public health importance. They underscore suboptimal compliance to parenteral artesunate dosing guidelines, a gap which may contribute to unfavourable outcomes, including death. Inadequate dosing may also contribute to treatment failure and potentially facilitate resistance.^10,11^ These findings call for strengthened monitoring and enforcement of compliance with malaria management guidelines.

The study has several strengths. It addresses an identified operational research priority with the potential to inform policy and practice. Conducted under the SORT IT programme,^12^ it benefited from an established framework for capacity building, rigorous data validation, and robust peer review. The study period spanned an entire year, capturing temporal variations in malaria incidence. We also adhered to the Strengthening the Reporting of Observational Studies in Epidemiology (STROBE) guidelines.^13^

The study also has some limitations. The main limitation was the absence of a qualitative component, which limited exploration of the underlying reasons why some children did not receive rapid diagnostic testing for malaria confirmation, why laboratory microscopy and other supportive investigations were underutilised in this tertiary facility, and why a substantial proportion of children received suboptimal or incomplete parenteral artesunate dosing. In addition, the study relied on routinely collected hospital records, which may be subject to incomplete documentation and limited ability to distinguish between prescription and actual administration of treatment. To limit this shortcoming, the team conducted regular data validation. Hospital discharge was used as a proxy for favourable outcome and does not directly measure parasitological cure. Furthermore, the study included children admitted with suspected severe malaria based on clinical criteria, and confirmatory microscopy was not performed; therefore, deaths cannot be attributed solely to malaria or treatment quality, as comorbidities and advanced disease at presentation could also have contributed. Finally, this analysis focused on inpatient parenteral artesunate administration and did not assess completion of oral ACT after discharge. These gaps highlight important areas for future operational research. Limitations notwithstanding, there are a number of policy and practice implications.

First, nearly one third of admitted children were malnourished, and this was significantly associated with unfavourable outcomes. This aligns with existing evidence that malnutrition exacerbates malaria severity, compromises immune response, and increases the risk of mortality, emphasising the need for heightened clinical vigilance in this vulnerable group.^14,15^ A ‘sick cohort’ effect may also be at play, as children reaching the tertiary referral facility are likely to represent more severe cases.

Second, we have identified important gaps in diagnostic practices. Although RDTs were routinely used for confirmatory malaria diagnosis, no microscopy was performed. Only one supportive laboratory investigation (blood sugar) was performed and that too in a limited number of children. Key investigations such as serum lactate, creatinine, and urine blood cells which are considered ‘standard of care’ in suspected severe malaria were not performed. This limited diagnostic capacity may compromise early detection of complications and optimal case management. Notably, the absence of parasite microscopy in all children is unexpected in a tertiary hospital. This test is valuable for assessing baseline and follow-up parasite densities while on treatment – an important prognostic and clinical marker of treatment response. It is also needed for malaria species identification and quality control of RDTs.

Third, compliance with treatment recommendation for parenteral artesunate was suboptimal at 57% but still higher when compared with Uganda where it was 18%.^11^ Although most children received parenteral artesunate, one third were administered incorrect weight-based doses and a subset of children admitted with suspected severe malaria did not receive the treatment at all – representing a ‘missed opportunity’ to act on preventable deaths. Notably, the majority of deaths occurred among children who did not receive artesunate in the recommended doses, suggesting that strengthening compliance might favourably impact survival. However, as the study included children with suspected severe malaria, deaths cannot be attributed solely to malaria or treatment quality, as other comorbidities and advanced disease at presentation may have contributed. The underlying reasons for this shortfall are unclear but may reflect broader health-system challenges, including limited staff capacity, inadequate supervision, documentation gaps, and possible stock-outs of essential diagnostics and drugs. Strengthening provider training and supervision to achieve 100% guideline compliance to artesunate use, coupled with a systematic audit of drug and diagnostic supply chains, should be considered as urgent first steps towards improving quality of care. A concrete suggestion would be to introduce standardised patient master cards for suspected severe malaria that include the core aspects of malaria case management where any deviation from the norm can be captured. This will also allow for a quarterly review of subset (or all) records and feedback on health facilities.

Finally, the peak months for malaria were identified as June and October, periods during which the hospital is likely to experience increased patient loads and strain on resources. Children presenting with factors associated with unfavourable outcomes (i.e., incorrect or incomplete artesunate dosing, malnutrition, and multiple convulsions) should receive targeted attention to reduce mortality.

## CONCLUSION

Compliance with parenteral artesunate dosing guidelines was suboptimal, likely contributing to preventable deaths and treatment failures, and may potentially facilitate artesunate resistance. Strengthened monitoring, mentorship, and enforcement of malaria management guidelines are urgently needed. Implementing continuing operational research is also a key strategy to monitor the impact of corrective measures. The findings from this study provide actionable insights for the Ministry of Health, hospital administrators, and health development partners.
